# The Chadwick Home Hospital, Liverpool

**Published:** 1887-07-09

**Authors:** 

**Affiliations:** Willoughby, Rugby


### THE EDITOR'S LETTER-BOX.

### THE CHADWICK HOME HOSPITAL, LIVERPOOL.

As the question often is asked, where a suitable Nursing Home, under the superintendence of a trained lady, can be had with moderate terms, and the patients be looked after and nursed by the lady herself, will you kindly bring to the notice of the public, through your valuable paper THE HOSPITAL, that such a home has been opened by Miss Chadwick, from the Royal Southern Hospital, at 98, Huskisson Street, Liverpool? Ladies, gentlemen, and children are received. The home is well situated on high ground, and is in easy access of the railway station. The rooms are large, warm, with every comfort. Patients can be attended by their own medical man. Medical, surgical, and obstetric admitted; mental, inebriate, or infectious cases not received. References to the profession given. Miss Chadwick has had great experience in her work, and is quite competent to undertake any of the above cases mentioned for treatment ; and I have no hesitation in saying that patients placed under her care will meet with that kindness and gentleness which is so essential to the sick and suffering.

SISTER OF THE GUILD OF ST. VERONICA.

Willoughby, Rugby.

